# Iminopyridine-Based Cobalt(II) and Nickel(II) Complexes: Synthesis, Characterization, and Their Catalytic Behaviors for 1,3-Butadiene Polymerization

**DOI:** 10.3390/polym8010012

**Published:** 2016-01-12

**Authors:** Quanquan Dai, Xiangyu Jia, Feng Yang, Chenxi Bai, Yanming Hu, Xuequan Zhang

**Affiliations:** Key Laboratory of Synthetic Rubber, Changchun Institute of Applied Chemistry, Chinese Academy of Sciences, Changchun 130022, China; qqdai@ciac.ac.cn (Q.D.); xiangyu_j@163.com (X.J.); yf18231@ciac.ac.cn (F.Y); xqzhang@ciac.ac.cn (X.Z.)

**Keywords:** iminopyridine, cobalt, nickel, 1,3-butadiene, polybutadiene

## Abstract

A series of iminopyridine ligated Co(II) (**1a**–**7a**) and Ni(II) (**1b**–**7b**) complexes were synthesized. The structures of complexes **3a**, **4a**, **5a**, **7a**, **5b**, and **6b** were determined by X-ray crystallographic analyses. Complex **3a** formed a chloro-bridged dimer, whereas **4a**, **5a**, and **7a**, having a substituent (**4a**, **5a**: CH_3_; **7a**: Br) at the 6-position of pyridine, producing the solid structures with a single ligand coordinated to the central metal. The nickel atom in complex **5b** features distorted trigonal-bipyramidal geometry with one THF molecule ligating to the metal center. All the complexes activated by ethylaluminum sesquichloride (EASC) were evaluated in 1,3-butadiene polymerization. The catalytic activity and selectivity were significantly influenced by the ligand structure and central metal. Comparing with the nickel complexes, the cobalt complexes exhibited higher catalytic activity and *cis*-1,4-selectivity. For both the cobalt and nickel complexes, the aldimine-based complexes showed higher catalyst activity than their ketimine counterparts.

## 1. Introduction

Conjugated diene polymerization is a long-standing research subject of considerable interest from the viewpoints of fundamental and practical perspectives. 1,3-butadiene (BD) polymerization is capable of producing polybutadienes (PBDs) with different isomeric forms such as *cis*-1,4, *trans*-1,4, 1,2-syndiotactic, 1,2-isotactic, and 1,2-atactic, and each exhibits different properties and consequently find diverse applications [1]. Due to the elastomeric character, *cis*-1,4-polybutadiene is one of the most important raw materials in rubber industry. Industrially, Ziegler–Natta catalysts based on Ti, Nd, Co, Ni, and other transition metals are used in the production of cis-1,4-PBDs [2,3,4].

Cobalt-based catalysts attract special interests as the microstructure of the product, including *cis*-1,4-PBD and syndiotactic 1,2-PBD, depending on the structure of the ligand coordinated to central metal. As a result, a variety of cobalt complexes have been designed and exploited for *cis*-1,4 and 1,2-selected polymerization, such as the cobalt halides and carboxylates, or the combination of alkylphosphines and pyridyl adducts as electron donors when activated by methylaluminoxane (MAO) [5,6]. Because of their ill-defined nature, these multi-site catalysts often produced polymers with broad molecular weight distribution, resulting in poor mechanical properties. In order to gain better control over molecular weight, molecular weight distribution, and, more importantly, stereoselectivity of the polymerization, academic and industrial research has focused on well-defined organometallic single-site catalysts. For example, four-coordinated (salen)cobalt(II) ([ONNO]^2−^) [7] and bis(salicylaldiminate)cobalt(II) [8], three-coordinated bis(imino)pyridine cobalt (II) [9], bis(benzimidazolyl)amine cobalt (II) [10], bis(benzimidazolyl) pyridine [11,12] cobalt(II), bis(thiazolinyl)pyridine cobalt(II) [13], 2-arylimino-6-(alcohol)pyridine/2-arylamino-6-(alcohol) pyridine cobalt(II) [14], and 3-aryliminomethyl-2-hydroxybenzaldehyde cobalt(II) [15] exhibited high activity and high *cis*-1,4 selectivity in 1,3-butadiene polymerization in combination with MAO or EASC. The nickel-based catalysts are also of particular interest in the production of high *cis*-1,4-PBD. For example, (salen)nickel(II) ([ONNO]^2−^) [16], nickel-tropolonoide, and nickel-1,3-propanedionate ([OO]^−^) [17] afforded polymers with high *cis*-1,4 content, whereas nickel dihalide complexes bearing neutral α-diimine ligands ([NN]) [16,18] or bis(imino)pyridine ligands ([NNN]) [17,19] were less active for the polymerization of 1,3-butadiene. Li *et al.* found that 2-arylimino-6-(alcohol)pyridine/2-arylamino-6-(alcohol)pyridine nickel (II) [14] produced the polymers with low molecular weight (*M*_n_ = 6000–9000) in high yields and the molecular weight distributions were somewhat broad (*M*_w_/*M*_n_ = 3.18–4.25), while the ligand environments did not influence the stereoregularity of the resulting polymers.

We have been interested in the synthesis of transition metal complexes to promote 1,3-butadiene polymerization over the past decades. For example, *cis*-1,4 selective polymerization of 1,3-butadiene was achieved by bis(imino)pyridine cobalt(II) complexes/MAO catalyst [9], and the incorporation of electron-withdrawing groups could enhance activity and selectivity simultaneously. Moreover, other catalyst systems usually focused on the steric and electronic effects of the substitutions at the iminoaryl rings on the catalytic activity and properties of resulting polymers. Even though some of these complexes based on the iminopyridine ligands have been reported for ethylene polymerization and oligomerization [20,21,22,23,24], iminopyridine cobalt(II) and nickel(II) complexes for 1,3-butadiene polymerization have not been investigated yet. In the present study, iminopyridine cobalt(II) and nickel(II) complexes were synthesized, and the influences of the substituents at the 6-position of the pyridine ring and in the imino bridge on catalytic activity and selectivity in 1,3-butadiene polymerization were investigated.

## 2. Materials and Methods

### 2.1. General Considerations and Materials

All the manipulations were carried out in a nitrogen atmosphere by using standard Schlenk techniques. 1,3-Butadiene (Jinzhou Petrochemical Company, Jinzhou, China) was purified by passing through two columns containing potassium hydroxide and active alumina. Hexane (Beijing Reagents Factory, Beijing, China) was refluxed over sodium benzophenone ketyl until the solution turned blue, and then distilled before use. Ethylaluminum sesquichloride (EASC) was purchased from Acros Chemicals (Geel, Belgium). CoCl_2_, NiBr_2_ and all the anilines were obtained from Alfa Aesar (Ward Hill, UK). Other chemicals were commercially available and used without further purification.

FTIR spectra were performed on a BRUKE Vertex-70 FTIR spectrometer (Bruker Optics, Ettlingen, Germany). Elemental analysis was performed using an elemental Vario EL spectrophotometer (Elementar, Hanau, Germany). The molecular weights (*M*_w_ and *M*_n_) and molecular weight distributions (*M*_w_/*M*_n_) of polymers were measured at 30 °C by gel permeation chromatography (GPC) equipped with a Waters 515HPLC pump, a series of four columns (HMW7THF, HMW6ETHF(two), HMW2THF) and a Waters 2414 refractive index detector (Waters, Massachusetts, UK). Tetrahydrofuran was used as an eluent at a flow rate of 1.0 mL/min.

X-ray crystallography measurements. Single crystals of **3a**, **4a**, **5a**, **7a**, **5b**, and **6b** suitable for X-ray diffraction were obtained by a slow diffusion of diethyl ether into their THF solution. Data collections were performed at −85 °C on a BRUKE SMART APEX diffractometer (Bruker, Bremen, Germany) with a CCD area detector, using graphite monochromated Mo K radiation (λ = 0.71073 Å). The determination of crystal class and unit cell parameters was carried out by the SMART program package. The raw frame data were processed using SAINT and SADABS to yield the reflection data file. The structures were solved by using SHELXTL program. Refinement was performed on F^2^ anisotropically for all non-hydrogen atoms by the full-matrix least-squares method. The hydrogen atoms were placed at the calculated positions and were included in the structure calculation without further refinement of the parameters.

### 2.2. Procedure for 1,3-Butadiene Polymerization

A typical procedure for the polymerization is as follows: a toluene solution of 1,3-butadiene (1.0 g, 1.85 × 10^−2^ mol) was added to a moisture free ampoule preloaded with complex **1a** (7.3 mg, 1.85 × 10^−5^ mol), and EASC (1.156 × 10^−3^ mol/mL, 0.4 mL) was then injected to initiate the polymerization at 20 °C. After 15 min, methanol was added to the system to quench the polymerization. The mixture was poured into a large quantity of methanol containing 2,6-di-tertbutyl-4-methylphenol (1.0 wt %) as a stabilizer. Via filtering and drying under vacuum at 40 °C, polybutadiene resulted at a constant weight (0.569 g, 56.9%).

### 2.3. Synthesis of Ligand (**L1–L7**)

#### 2.3.1. 2,6-Bis(1-methylethyl)-*N*-(2-pyridinylmethylene)phenylamine (**L1**)

Ligand **L1** was prepared according to the reported method [21]. Pyridine-2-carbaldehyde (2.0 g, 0.0187 mol) was dissolved in 30 mL of methanol, then 2,6-di(1-methylethyl)phenylamine (3.55 g, 0.02 mol), and a few drops of formic acid were subsequently added. This mixture was refluxed for 12 h. The solvent was evaporated *in vacuo*. The remaining crude product was dissolved in *n*-pentane, and the solution was dried with Na_2_SO_4_. After filtration, the compound **L1** was crystallized from *n*-pentane at −25 °C, producing yellow crystals. Yield: 3.75 g (75.4%). ^1^H NMR (400 MHz, CDCl_3_, δ, ppm): 1.17 (d, 12 H, CHMe_2_), 2.97 (m, 2 H, CHMe_2_), 7.11–7.28 (m, 3 H, H_phenyl_), 7.41 (t, 1 H, H_pyridine,5_), 7.85 (t, 1 H, H_pyridine,4_), 8.26 (d, 1 H, H_pyridine,3_), 8.31 (s, 1 H, CH=N), 8.72 (d, 1 H, H_pyridine,6_). ^13^C NMR (100 MHz, CDCl_3_, δ, ppm): 162.80 (CH=N), 149.51, 148.22, 137.05, 136.54, 125.12, 124.30, 122.88, 122.57, 121.14, 27.77 (CHMe_2_), 23.27 (CHMe_2_). IR (KBr, cm^−1^): 2959, 1633, 1586, 1470, 1385, 808, 779, 754. Anal. Calcd. For C_18_H_22_N_2_: C, 81.16; H, 8.32; N, 10.52. Found: C, 81.59; H, 7.96; N, 10.45.

#### 2.3.2. 2,6-Bis(1-methylethyl)-*N*-[1-(2-pyridinyl)-ethylidene]phenylamine (**L2**)

The procedure as above in (4.2.1) by using 2-acetyl-pyridine (1.0 g, 0.0083 mol) and 2,6-di(1-methylethyl)phenylamine (1.77 g, 0.01 mol) produced **L2** as yellow compound crystallized from methanol at −25 °C in 82.2% yield. ^1^H NMR (400 MHz, CDCl_3_, δ, ppm): 1.16 (d, 12 H, CHMe_2_), 2.21 (s, 3 H, C(CH_3_)=N), 2.75 (m, 2 H, CHMe_2_), 7.07–7.15 (m, 3 H, H_phenyl_), 7.39 (t, 1 H, H_pyridine,5_), 7.79 (t, 1 H, H_pyridine,4_), 8.35 (t, 1 H, H_pyridine,3_) 8.69 (t, 1 H, H_pyridine,6_). ^13^C NMR (100 MHz, CDCl_3_, δ, ppm): 166.77 (CMe=N), 156.29, 148.39, 146.25, 136.25, 135.58, 124.57, 123.40, 122.80, 121.11, 28.06 (CHMe_2_), 23.03 (CHMe_2_), 22.69 (CHMe_2_), 17.11 (C(CH3)=N). IR (KBr, cm^−1^): 2958, 1637, 1585, 1566, 1363, 933, 824, 775, 743. Anal. Calcd. For C_19_H_24_N_2_: C, 81.38; H, 8.63; N, 9.99. Found: C, 81.78; H, 8.24; N, 9.98.

#### 2.3.3. 2,6-Bis(1-methylethyl)-*N*-(phenyl-2-pyridinylmethylene)phenylamine (**L3**)

Phenyl-2-pyridinylmethanone (2.0 g, 0.01 mol) was dissolved in 30 mL of methanol, then 2,6-di(1-methylethyl)phenylamine (1.95 g, 0.011 mol) and a few drops of concentrated H_2_SO_4_ were added. This mixture was refluxed for 12 h. The solvent was evaporated *in vacuo*. The yellow raw product was dissolved in 50 mL of dichloromethane, filtered, washed with 50 mL of water and dried with Na_2_SO_4_. After filtration and solvent evaporation, the yellow residue was purified by crystallization from methanol at −25 °C, affording yellow, needle-like crystals in 46.7% yield. ^1^H NMR (400 MHz, CDCl_3_, δ, ppm): 0.89 (d, 3 H, CHMe_2_), 0.96 (d, 3 H, CHMe_2_), 1.13 (d, 6H, CHMe_2_), 2.86 (m, 1 H, CHMe_2_), 2.96 (m, 1 H, CHMe_2_), 6.96-7.21 (m, 6 H, H_arom_), 7.44 (m, H_arom_), 7.81 (m, H_arom_), 8.23 (d, H_arom_), 8.61 (d, H_arom_). ^13^C NMR (100 MHz, CDCl_3_, δ, ppm): 165.26 (C(Phenyl)=N), 164.04, 157.52, 154.25, 148.91, 148.59, 145.91, 138.17, 136.36, 135.31, 134.69, 130.36, 129.39, 129.06, 128.03, 127.33, 123.46, 123.09, 122.41, 28.15 (CHMe_2_), 23.74 (CHMe_2_), 23.64 (CHMe_2_), 21.81 (CHMe_2_), 21.71(CHMe_2_). IR (KBr, cm^−1^): 2955, 1616, 1584, 1380, 831, 799, 774. Anal. Calcd. For C_24_H_26_N_2_: C, 84.17; H, 7.65; N, 8.18. Found: C, 84.65; H, 8.02; N, 7.33.

#### 2.3.4. 2,6-Bis(1-methylethyl)-*N*-[(6-methyl-2-pyridinyl)-methylene]phenylamine (**L4**)

The procedure as above in (4.2.1) by using 6-methyl-pyridine-2-carbaldehyde (1.0 g, 0.0083 mol) and 2,6-di(1-methylethyl)phenylamine (1.77g, 0.01 mol) produced **L4** as yellow compound crystallized from methanol at −25 °C in 92.6% yield. ^1^H NMR (400 MHz, CDCl_3_, δ, ppm): 1.16 (d, 12 H, CHMe_2_), 2.64 (s, 3 H, H_pyridine_,_6-Me_), 2.97 (m, 2 H, CHMe_2_), 7.09–7.28 (m, 3 H, H_phenyl_), 7.28 (d, 1 H, H_pyridine,5_), 7.72 (t, 1 H, H_pyridine,4_), 8.07 (d, 1 H, H_pyridine,3_), 8.27 (s, 1 H, CH=N). ^13^C NMR (100 MHz, CDCl_3_, δ, ppm): 163.06 (CH=N), 158.28, 153.69, 148.33, 136.98, 136.67, 124.78, 124.14, 122.78, 118.21, 27.72 (CHMe_2_), 24.18 (C_pyridine, 6-Me_), 23.23 (CHMe_2_). IR (KBr, cm^−1^): 2959, 1642, 1588, 1456, 1318, 856, 795, 748. Anal. Calcd. For C_19_H_24_N_2_: C, 81.38; H, 8.63; N, 9.99. Found: C, 81.26; H, 8.85; N, 9.89.

#### 2.3.5. 2,6-Bis(1-methylethyl)-*N*-[1-(6-methyl-2-pyridinyl)-ethylidene]phenylamine (**L5**)

The procedure as above in (4.2.1) by using 6-methyl-2-acetylpyridine (2.0 g, 0.0148 mol) and 2,6-di(1-methylethyl)phenylamine (2.66 g, 0.015 mol) produced **L5** as yellow compound crystallized from methanol at −25 °C in 89.2% yield. ^1^H NMR (400 MHz, CDCl_3_, δ, ppm): 1.13 (d, 12 H, CHMe_2_), 2.20 (s, 3 H, C(CH_3_)=N), 2.62 (s, 3 H, H_pyridine, 6-Me_), 2.75 (m, 2 H, CHMe_2_), 7.06–7.24 (m, 3 H, H_phenyl_), 7.24 (d, 1 H, H_pyridine,5_), 7.68 (t, 1 H, H_pyridine,4_), 8.14 (d, 1 H, H_pyridine,3_). ^13^C NMR (100 MHz, CDCl_3_, δ, ppm): 167.18 (CH=N), 157.16, 155.77, 146.47, 136.35, 135.63, 124.00, 123.27, 122.77, 118.04, 28.04 (CHMe_2_), 24.37 (C_pyridine, 6-Me_), 23.05 (CHMe_2_), 22.73 (CHMe_2_), 17.11 (C(CH_3_)=N). IR (KBr, cm^−1^): 2961, 1643, 1586, 1458, 1192, 892, 763, 742. Anal. Calcd. For C_20_H_26_N_2_: C, 81.59; H, 8.90; N, 9.51. Found: C, 81.83; H, 9.33; N, 8.84.

#### 2.3.6. 2,6-Bis(1-methylethyl)-*N*-[(6-bromo-2-pyridinyl)-methylene]phenylamine (**L6**)

6-Bromo-pyridine-2-carbaldehyde (1.0 g, 0.005 mol) was dissolved in 30 mL of methanol, then 2,6-di(1-methylethyl)phenylamine (1.06 g, 0.006 mol), and a few drops of formic acid were subsequently added. This mixture was stirred at room temperature, a yellow precipitate was formed after 10 min. The mixture was stirred for another 5 h, then the precipitation was collected and washed twice with cold methanol, yielding the analytically pure compound **L6** ( 1.64 g, 95.4%) as a yellow powder. ^1^H NMR (400 MHz, CDCl_3_, δ, ppm): 1.15 (d, 12 H, CHMe_2_), 2.92 (m, 2 H, CHMe_2_), 7.11-7.18 (m, 3 H, H_phenyl_), 7.60 (d, 1 H, H_pyridine,3_), 7.71 (t, 1 H, H_pyridine,4_), 8.24 (s, 1 H, CH=N), 8.26 (d, 1 H, H_pyridine,5_). ^13^C NMR (100 MHz, CDCl_3_, δ, ppm): 161.31 (CH=N), 155.28, 147.75, 141.69, 138.83, 136.85, 129.64, 124.53, 122.90, 119.71, 27.78 (CHMe_2_), 23.22 (CHMe_2_). IR (KBr, cm^−1^): 2959, 1644, 1574, 1362, 1185, 809, 796, 763. Anal. Calcd. For C_18_H_21_BrN_2_: C, 62.61; H, 6.13; N, 8.11. Found: C, 61.59; H, 5.81; N, 8.64.

#### 2.3.7. 2,6-Bis(1-methylethyl)-*N*-[1-(6-bromo-2-pyridinyl)-ethylidene]phenylamine (**L7**)

The procedure as above in (4.2.6) using 2-bromo-6-acetylpyridine (1.0 g, 0.005 mol) and 2,6-di(1-methylethyl)phenylamine (0.975 g, 0.0055 mol) produced **L7** as yellow compound in 74.6% yield. ^1^H NMR (400 MHz, CDCl_3_, δ, ppm): 1.12–1.15 (m, 12 H, CHMe_2_), 2.18 (s, 3 H, C(Me)=N), 2.69 (m, 2 H, CHMe_2_), 7.07–7.16 (m, 3 H, H_phenyl_), 7.56 (d, 1 H, H_pyridine,3_), 7.65 (t, 1 H, H_pyridine,4_), 8.33 (d, 1 H, H_pyridine,5_). ^13^C NMR (100 MHz, CDCl_3_, δ, ppm): 165.72 (C(Me)=N), 157.22, 145.94, 140.79, 138.56, 135.44, 131.56, 128.98, 123.61, 122.83, 120.26, 119.83, 28.08 (CHMe_2_), 23.01 (CHMe_2_), 22.64 (CHMe_2_), 17.07 (C(Me)=N). IR (KBr, cm^−1^): 2960, 1640, 1551, 1436, 1361, 1306, 834, 753, 734. Anal. Calcd. For C_19_H_23_BrN_2_: C, 63.51; H, 6.45; N, 7.80. Found: C, 62.94; H, 6.62; N, 8.10.

### 2.4. Preparation of Cobalt and Nickel Complexes

#### 2.4.1. (2,6-Bis(1-methylethyl)-*N*-(2-pyridinylmethylene)phenylamine)cobalt(II) dichloride, **1a**

A mixture of **L1** (0.2 g, 0.00075 mol) and anhydrous CoCl_2_ (0.1 g, 0.00075 mol) were added to a flask containing 5 mL THF. The mixture was stirred at room temperature for 24 h, a green suspension was formed. Diethyl ether was added and a suspension formed. The precipitate was collected by filtration and washed with 4 × 5 mL heptane. The desired product (0.21 g, 70.7%) was obtained after dried *in vacuo* at 40 °C. IR (KBr, cm^−1^): 2968, 1625, 1593, 1465, 1383, 806, 775, 762. Anal. Calcd. For C_18_H_22_Cl_2_CoN_2_: C, 54.56; H, 5.60; N, 7.07. Found: C, 53.86; H, 6.01; N, 7.26.

#### 2.4.2. (2,6-Bis(1-methylethyl)-*N*-[1-(2-pyridinyl)-ethylidene]phenylamine)cobalt(II) dichloride, **2a**

Procedure similar for **2a** was adopted by using **L2**, and CoCl_2_ produced **2a** as a green powder in 82.3% yield. IR (KBr, cm^−1^): 2968, 1612, 1591, 1572, 1373, 939, 846, 783, 753. Anal. Calcd. For C_19_H_24_Cl_2_CoN_2_: C, 55.63; H, 5.90; N, 6.83. Found: C, 56.03; H, 6.17; N, 6.65.

#### 2.4.3. (2,6-Bis(1-methylethyl)-*N*-(phenyl-2-pyridinylmethylene)phenylamine) cobalt(II) dichloride, **3a**

Procedure similar for **3a** was adopted by using **L3**, and CoCl_2_ produced **3a** as a green powder in 52.8% yield. IR (KBr, cm^−1^): 2974, 1609, 1570, 1384, 800, 778, 765. Anal. Calcd. For C_24_H_26_Cl_2_CoN_2_: C, 61.03; H, 5.55; N, 5.93. Found: C, 61.24; H, 5.75; N, 6.02.

#### 2.4.4. (2,6-Bis(1-methylethyl)-*N*-[(6-methyl-2-pyridinyl)-methylene]phenylamine) cobalt(II) dichloride, **4a**

Procedure similar for **4a** was adopted by using **L4**, and CoCl_2_ produced **4a** as a green powder in 92.7% yield. IR (KBr, cm^−1^): 2966, 1633, 1596, 1466, 1328, 866, 802, 742. Anal. Calcd. For C_19_H_24_Cl_2_CoN_2_: C, 55.63; H, 5.90; N, 6.83. Found: C, 55.37; H, 6.22; N, 7.29.

#### 2.4.5. (2,6-Bis(1-methylethyl)-*N*-[1-(6-methyl-2-pyridinyl)-ethylidene]phenylamine) cobalt(II) dichloride, **5a**

Procedure similar for **5a** was adopted by using **L5**, and CoCl_2_ produced **5a** as a green powder in 86.4% yield. IR (KBr, cm^−1^): 2963, 1618, 1593, 1460, 1196, 888, 779, 740. Anal. Calcd. For C_20_H_26_Cl_2_CoN_2_: C, 56.62; H, 6.18; N, 6.60. Found: C, 56.80; H, 6.03; N, 6.58.

#### 2.4.6. (2,6-Bis(1-methylethyl)-*N*-[(6-bromo-2-pyridinyl)-methylene]phenylamine) cobalt(II) dichloride, **6a**

Procedure similar for **6a** was adopted by using **L6**, and CoCl_2_ produced **6a** as a green powder in 91.2% yield. IR (KBr, cm^−1^): 2961, 1630, 1584, 1365, 1177, 813, 803, 765. Anal. Calcd. For C_18_H_21_BrCl_2_CoN_2_: C, 45.50; H, 4.46; N, 5.90. Found: C, 44.72; H, 4.77; N, 6.28.

#### 2.4.7. (2,6-Bis(1-methylethyl)-*N*-[1-(6-bromo-2-pyridinyl)-ethylidene]phenylamine) cobalt(II) dichloride, **7a**

Procedure similar for **7a** was adopted by using **L7**, and CoCl_2_ produced **7a** as a green powder in 88.1% yield. IR (KBr, cm^−1^): 2966, 1619, 1555, 1445, 1367, 1311, 859, 755, 730. Anal. Calcd. For C_19_H_23_BrCl_2_CoN_2_: C, 46.65; H, 4.74; N, 5.73. Found: C, 47.09; H, 4.52; N, 6.27.

#### 2.4.8. (2,6-Bis(1-methylethyl)-*N*-(2-pyridinylmethylene)phenylamine)nickel(II) dibromide, **1b**

To a suspension of NiBr_2_(dme) (0.11 g, 0.00036 mol) in 5 mL, CH_2_Cl_2_ was added into the solution of ligand **L1** (0.1 g, 0.00036 mol) in 5 mL CH_2_Cl_2_. The orange-red reaction mixture was formed and stirred for 24 h at room temperature, and the precipitate was collected by filtration, washed with pentane (2 × 10 mL), and dried under vacuum to produce complex **1b** as an orange-red powder. Yield: 0.15 g (83.6%). IR (KBr, cm^−1^): 2964, 1630, 1596, 1387, 802, 775, 760. Anal. Calcd. For C_18_H_22_Br_2_N_2_Ni: C, 44.59; H, 4.57; N, 5.78. Found: C, 44.69; H, 4.77; N, 5.44.

#### 2.4.9. (2,6-Bis(1-methylethyl)-*N*-[1-(2-pyridinyl)-ethylidene]phenylamine)nickel(II) dibromide, **2b**

Procedure similar for **2b** was adopted by using **L2**, and NiBr_2_(dme) produced **2b** as a green powder in 91.7% yield. IR (KBr, cm^−1^): 2966, 1612, 1591, 1571, 1373, 940, 816, 781, 752. Anal. Calcd. For C_19_H_24_Br_2_N_2_Ni: C, 46.74; H, 4.85; N, 5.61. Found: C, 46.99; H, 4.81; N, 5.87.

#### 2.4.10. (2,6-Bis(1-methylethyl)-*N*-(phenyl-2-pyridinylmethylene)phenylamine) nickel(II) dibromide, **3b**

Procedure similar for **3b** was adopted by using **L3**, and NiBr_2_(dme) produced **3b** as a green powder in 47.5% yield. IR (KBr, cm^−1^): 2964, 1632, 1596, 1379, 801, 776, 741. Anal. Calcd. For C_24_H_26_Br_2_N_2_Ni: C, 51.38; H, 4.67; N, 4.99. Found: C, 51.06; H, 4.37; N, 5.12

#### 2.4.11. (2,6-Bis(1-methylethyl)-*N*-[(6-methyl-2-pyridinyl)-methylene]phenylamine) nickel(II) dibromide, **4b**

Procedure similar for **4b** was adopted by using **L4**, and NiBr_2_(dme) produced **4b** as a green powder in 76.7% yield. IR (KBr, cm^−1^): Ni: 2962, 1632, 1597, 1467, 1330, 867, 800, 741. Anal. Calcd. For C_19_H_24_Br_2_N_2_Ni: C, 45.74; H, 4.85; N, 5.61. Found: C, 45.85; H, 5.09; N, 4.62

#### 2.4.12. (2,6-Bis(1-methylethyl)-*N*-[1-(6-methyl-2-pyridinyl)-ethylidene]phenylamine) nickel(II) dibromide, **5b**

Procedure similar for **5b** was adopted by using **L5**, and NiBr_2_(dme) produced **5b** as a green powder in 83.4% yield. IR (KBr, cm^−1^): Ni: 2965, 1617, 1595, 1461, 1196, 890, 779, 738. Anal. Calcd. For C_20_H_26_Br_2_N_2_Ni: C, 46.83; H, 5.11; N, 5.46. Found: C, 47.24; H, 5.34; N, 5.71

#### 2.4.13. (2,6-Bis(1-methylethyl)-*N*-[(6-bromo-2-pyridinyl)-methylene]phenylamine) nickel(II) dibromide, **6b**

Procedure similar for **6b** was adopted using **L6**, and NiBr_2_(dme) produced **6b** as a green powder in 93.6% yield. IR (KBr, cm^−1^): 2961, 1626, 1585, 1365, 1175, 810, 800, 763. Anal. Calcd. For C_18_H_21_Br_3_N_2_Ni: C, 38.35; H, 3.75; N, 4.97. Found: C, 37.86; H, 3.94; N, 5.12.

#### 2.4.14. (2,6-Bis(1-methylethyl)-*N*-[1-(6-bromo-2-pyridinyl)-ethylidene]phenylamine) nickel(II) dibromide, **7b**

Procedure similar for **7b** was adopted by using **L7**, and NiBr_2_(dme) produced **7b** as a green powder in 85.1% yield. IR (KBr, cm^−1^): 2965, 1613, 1553, 1440, 1369, 1315, 828, 764, 733. Anal. Calcd. For C_19_H_23_Br_3_N_2_Ni: C, 39.49; H, 4.01; N, 4.85. Found: C, 40.10; H, 3.71; N, 4.99.

## 3. Results and Discussions

### 3.1. Synthesis and Characterization of Iminopyridine Ligands (L1–L7) and Their Cobalt(II) and Nickel(II) Complexes

**Scheme 1:**
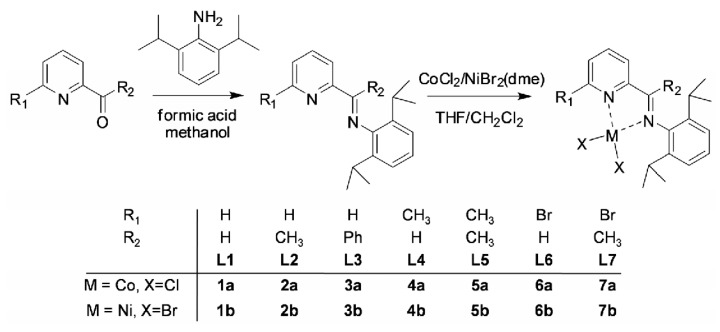
Synthesis of cobalt complexes **1a**–**7a** and nickel complexes **1b**–**7b**.

The iminopyridine ligands **L1**, **L2**, **L4**-**L7** were prepared by the condensation of 2-pyridinecarboxaldehydes or 2-pyridylketones with 2,6-di(1-methylethyl)phenylamine in the presence of a catalytic amount of formic acid in methanol (Scheme 1). Ligand **L3** was synthesized with a few drops of concentrated H_2_SO_4_ as catalyst, and anhydrous Na_2_SO_4_ was used to remove the water formed during the reaction according to the literature [21]. All the ligands were identified by FT-IR, elemental analysis, and NMR spectra. Cobalt complexes **1a**–**7a** were prepared by the reaction of anhydrous cobalt(II) chloride with the corresponding ligand in THF. All of the complexes were isolated as green air-stable powders in high yields. Nickel complexes **1b**–**7b** were prepared by the reaction of the corresponding ligand with NiBr_2_(dme) in CH_2_Cl_2_. The structures of these complexes were determined by FT-IR spectra and elemental analysis. The structures of complexes **3a**, **4a**, **5a**, **7a**, **5b**, and **6b** were further characterized by X-ray crystallographic analysis.

### 3.2. Crystal Structure of Complexes

Single crystals of **3a**, **4a**, **5a**, **7a**, **5b**, and **6b** suitable for X-ray diffraction analysis were obtained by crystallization from their THF or diethyl ether solutions. The crystal data together with the data collection and structure refinement parameters are presented in Table 1. Selected bond lengths and angles for cobalt complexes (**3a**, **4a**, **5a** and **7a**) are given in Table 2. Selected bond lengths and angles for nickel complexes (**5b** and **6b**) are given in Table 3.

**Table 1:** Crystal data and data collection parameters of cobalt and nickel complexes.

	3a·Et_2_O	4a	5a	7a	5b·THF	6b
Formula	C_52_H_62_Cl_4_Co_2_N_4_O	C_19_H_24_Cl_2_CoN_2_	C_20_H_26_Cl_2_CoN_2_	C_19_H_23_BrCl_2_CoN_2_	C_24_H_34_Br_2_N_2_NiO	C_36_H_42_Br_6_N_4_Ni_2_
Molecular weight	1,018.72	410.23	424.26	489.13	585.06	1,127.62
Wavelength (Å)	0.71073	0.71073	0.71073	0.71073	0.71073	0.71073
Crystal system	Monoclinic	Monoclinic	Monoclinic	Monoclinic	Monoclinic	Monoclinic
Space group	P2_1_/c	P2_1_/c	P2_1_/n	P2_1_/c	P2_1_/c	P2_1_/c
a (Å)	9.1341 (8)	10.1638 (6)	8.9107 (8)	16.6693 (12)	10.6823 (5)	10.336 (2)
b (Å)	16.4268 (15)	19.5791 (11)	16.0155 (14)	10.4115 (7)	18.6756 (8)	19.755 (4)
c (Å)	18.2838 (16)	20.5215 (11)	14.5532 (13)	12.4751 (9)	14.7019 (7)	20.644 (4)
α (deg)	90.00	90.00	90.00	90.00	90.00	90.00
β (deg)	103.371 (1)	95.071 (1)	90.034 (1)	106.366 (1)	109.092 (1)	93.835 (4)
γ (deg)	90.00	90.00	90.00	90.00	90.00	90.00
V (Å^3^)	2,669.0 (4)	4067.8 (4)	2,076.9 (3)	2,077.4 (3)	2,771.7 (2)	4,205.8 (14)
Z	2	8	4	4	4	4
D_calcd_ (Mg/m^3^)	1.268	1.340	1.357	1.564	1.402	1.781
Absorp coeff (mm^−1^)	0.86	1.11	1.09	3.01	3.60	6.625
F (000)	1,064	1704	884	988	1192	2208
Crystal size (mm)	0.28 × 0.13 × 0.09	0.25 × 0.21 × 0.09	0.28×0.17 × 0.10	0.21×0.12 × 0.07	0.27 ×0.13 × 0.10	0.25×0.16 × 0.11
θ Range (deg)	2.3–26.0	2.3–24.9	2.5–24.5	2.6–26.0	2.3–26.0	2.2–24.5
No. of reflns collected	15,743	23,659	15,347	14,498	20,676	26,672
No. of indep reflns	5243	7,178	4,071	4,034	5,455	8,310
No. of data/restraints/params	5,243/7/313 (*R*_int_ = 0.026)	7,178/0/443 (*R*_int_ = 0.042)	4,071/0/232 (*R*_int_ = 0.038)	4,034/0/231 (*R*_int_ = 0.023)	5,455/0/277 (*R*_int_ = 0.024)	8,310/0/441 (*R*_int_ = 0.055)
GOF on F^2^	1.05	0.98	1.01	1.04	1.04	0.97
R_1_ (1 > 2sigma(1))	0.045	0.038	0.036	0.030	0.025	0.039
wR_2_	0.145	0.090	0.090	0.083	0.068	0.083

**Table 2:** Selected bond distances (Å) and angles (°) of cobalt complexes **3a**, **4a**, **5a** and **7a**.

3a		4a		5a		7a	
Bond lengths							
Co1–N1	2.099 (2)	Co1–N1	2.181 (2)	Co1–N1	2.0439 (18)	Co1–N1	2.0667 (18)
Co1–N2	2.128 (2)	Co1–N2	2.089 (2)	Co1–N2	2.0498 (18)	Co1–N2	2.0514 (17)
Co1–Cl1	2.2830 (9)	Co1–Cl1	2.2769 (8)	Co1–Cl1	2.2071 (7)	Co1–Cl1	2.2079 (7)
Co1–Cl2	2.3607 (8)	Co1–Cl2	2.4428 (8)	Co1–Cl2	2.2148 (7)	Co1–Cl2	2.2235 (7)
Co1–Cl2A	2.4372 (8)	Co2–N4	2.097 (2)	N2–C7	1.290 (3)	N2–C8	1.451 (3)
Co1A–Cl2	2.4371 (8)	Co2–N3	2.182 (2)	N2–C9	1.448 (3)	N2–C6	1.285 (3)
C5–C6	1.493 (4)	Co2–Cl3	2.2709 (8)	C5–C7	1.492 (3)	C5–C6	1.492 (3)
N2–C6	1.301 (4)	Co2–Cl4	2.4433 (8)	C7–C8	1.490 (3)	C6–C7	1.489 (3)
N2–C13	1.454 (4)	N2–C8	1.442 (3)	C1–C6	1.491 (3)	Br1–C1	1.888 (3)
C6–C7	1.489 (4)	N2–C6	1.271 (3)				
		N4–C27	1.438 (3)				
		N4–C25	1.272 (3)				
Bond angles							
N1–Co1–N2	76.36 (9)	N1–Co1–N2	77.69 (8)	N1–Co1–N2	80.68 (7)	N2–Co1–N1	80.14 (7)
N1–Co1–Cl1	92.08 (7)	N1–Co1–Cl1	91.41 (6)	N1–Co1–Cl1	118.90 (6)	N1–Co1–Cl1	118.64 (6)
N2–Co1–Cl1	117.17 (7)	N2–Co1–Cl1	111.97 (6)	N2–Co1–Cl1	115.04 (6)	N2–Co1–Cl1	111.66 (5)
N1–Co1–Cl2	164.04 (8)	N1–Co1–Cl2	171.60 (6)	N1–Co1–Cl2	107.53 (5)	N2–Co1–Cl2	117.93 (5)
N2–Co1–Cl2	98.55 (7)	N2–Co1–Cl2	96.91 (6)	N2–Co1–Cl2	114.54 (6)	N1–Co1–Cl2	110.67 (5)
Cl1–Co1–Cl2	103.60 (3)	Cl1–Co1–Cl2	96.61 (3)	Cl1–Co1–Cl2	115.44 (3)	Cl1–Co1–Cl2	113.88 (3)
N1–Co1–Cl2A	85.08 (7)	N4–Co2–N3	77.66 (8)	C7–N2–C9	120.43 (19)	N1–C1–Br1	116.75 (17)
N2–Co1–Cl2A	125.21 (7)	N4–Co2–Cl3	114.12 (6)	N1–C5–C7	115.52 (19)	C2–C1–Br1	119.39 (19)
Cl1–Co1–Cl2A	114.55 (3)	N3–Co2–Cl3	94.45 (6)	C4–C5–C7	122.7 (2)	C7–C6–C5	118.39 (19)
Cl2–Co1–Cl2A	85.73 (3)	N4–Co2–Cl4	96.58 (6)	C8–C7–C5	118.6 (2)	C6–N2–C8	120.24 (18)
N2–C6–C7	125.8 (3)	N3–Co2–Cl4	169.32 (6)	N2–C7–C8	124.9 (2)	N2–C6–C7	125.0 (2)
C6–N2–C13	118.9 (2)	Cl3–Co2–Cl4	96.12 (3)	N1–C1–C6	116.7 (2)	N2–C6–C5	116.58 (19)
N2–C6–C5	115.7 (3)	N2–C6–C5	120.4 (2)	N2–C7–C5	116.4 (2)		

**Table 3:** Selected bond distances (Å) and angles (°) of nickel complexes **5b** and **6b**.

5b		6b			
Bond lengths					
Ni1–N2	2.0383 (16)	Ni1–N2	2.049 (4)	Br1–Ni1A	2.4770 (8)
Ni1–N1	2.0677 (17)	Ni1–N1	2.092 (3)	Br3–C1	1.887 (5)
Ni1–O1	2.1478 (15)	Ni1–Br2	2.4153 (8)	Br5–Ni2A	2.5076 (8)
Ni1–Br1	2.4376 (3)	Ni1–Br1A	2.4770 (8)	Br6–C19	1.875 (5)
Ni1–Br2	2.4602 (3)	Ni1–Br1	2.5230 (8)		
C5–C7	1.492 (3)	Ni2–N4	2.038 (3)		
C7–C8	1.494 (3)	Ni2–N3	2.080 (3)		
N2–C7	1.286 (3)	Ni2–Br4	2.4214 (9)		
N2–C9	1.445 (2)	Ni2–Br5	2.4777 (8)		
C1–C6	1.489 (3)	Ni2–Br5A	2.5075 (8)		
Bond angles					
N2–Ni1–N1	80.22 (6)	N2–Ni1–N1	80.27 (13)	N4–Ni2–Br4	107.02 (10)
N2–Ni1–Br1	106.94 (5)	N2–Ni1–Br2	107.62 (9)	N3–Ni2–Br4	90.38 (10)
N1–Ni1–Br1	91.16 (5)	N1–Ni1–Br2	90.87 (9)	N4–Ni2–Br5	104.32 (10)
N2–Ni1–Br2	111.61 (5)	N2–Ni1–Br1A	104.84 (10)	N3–Ni2–Br5	90.36 (10)
N1–Ni1–Br2	90.48 (5)	N1–Ni1–Br1A	90.92 (9)	Br4–Ni2–Br5	148.29 (3)
Br1–Ni1–Br2	141.124 (13)	Br2–Ni1–Br1A	147.32 (3)	N4–Ni2–Br5A	100.41 (9)
C5–C7–C8	118.23 (17)	N2–Ni1–Br1	100.45 (9)	N3–Ni2–Br5A	176.73 (10)
N2–C7–C8	125.39 (19)	N1–Ni1–Br1	176.28 (9)	Br4–Ni2–Br5A	92.55 (2)
N1–C1–C6	118.41 (19)	Br2–Ni1–Br1	92.38 (2)	Br5–Ni2–Br5A	86.39 (2)
C2–C1–C6	120.9 (2)	Br1A–Ni1–Br1	85.37 (2)	N2–C6–C5	120.1 (4)
C7–N2–C9	119.55 (16)	N4–Ni2–N3	80.09 (13)	N1–C1–Br3	117.2 (3)

**Figure 1:**
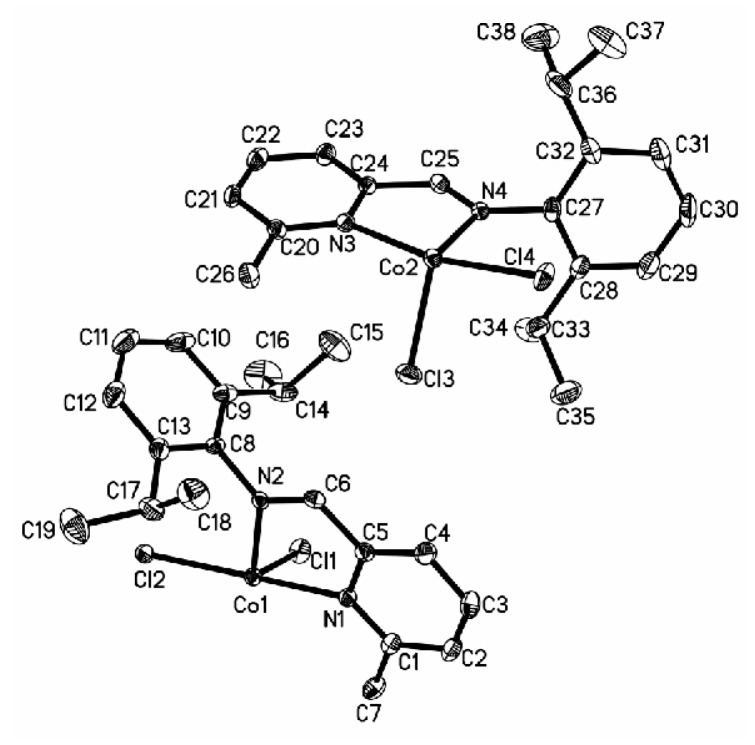
ORTEP view of complex **4a**, drawn at 35% of probability. Hydrogen atoms were omitted for clarity.

**Figure 2:**
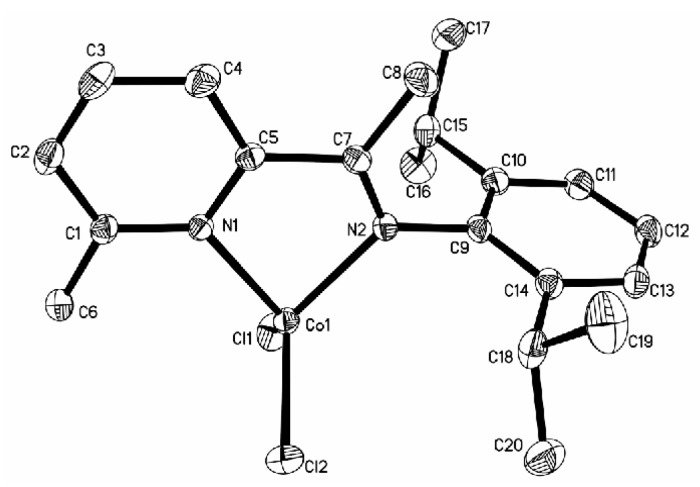
ORTEP view of complex **5a**, drawn at 35% of probability. Hydrogen atoms were omitted for clarity.

**Figure 3:**
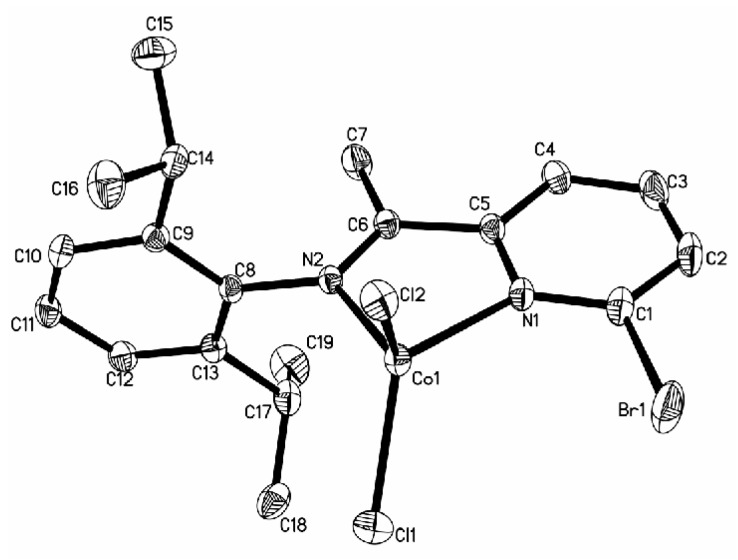
ORTEP view of complex **7a**, drawn at 35% of probability. Hydrogen atoms were omitted for clarity.

**Figure 4:**
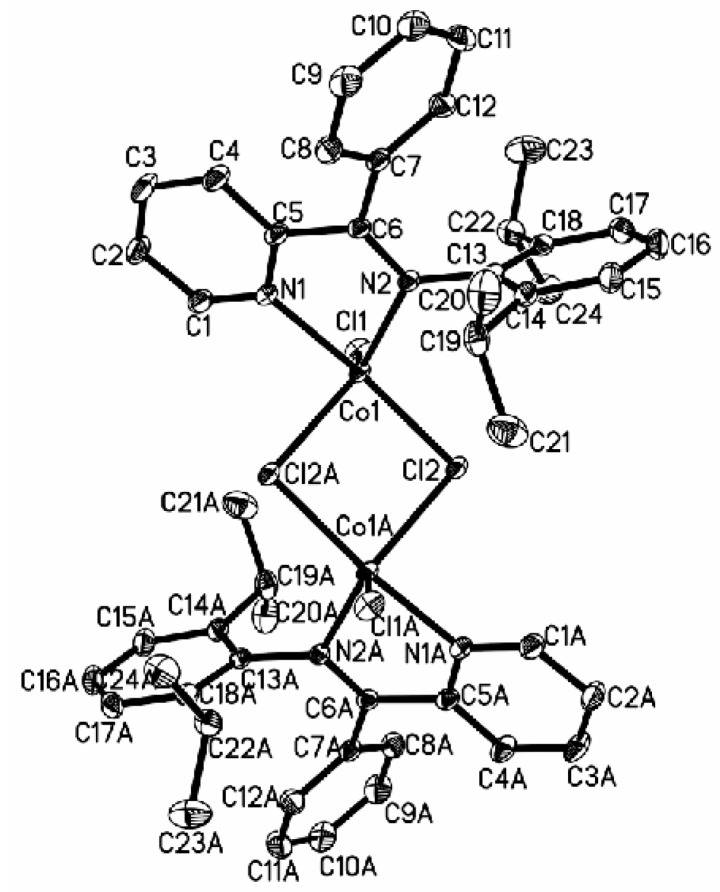
ORTEP view of complex **3a**, drawn at 35% of probability. Hydrogen atoms and one **Et_2_O** molecule were omitted for clarity.

Cobalt complexes **4a**, **5a**, and **7a** with a substituent (**4a**: CH_3_; **5a**: CH_3_; **7a**: Br) at the 6-position of the pyridine ring produced the solid structures with a single ligand coordinated to the central metal. The asymmetric unit of **4a** contains two independent, closely resembling molecules (Figure 1), whereas only one molecule is found in the asymmetric unit of complexes **5a** (Figure 2) and **7a** (Figure 3). In complex **4a** crystallizing in a monoclinic space group P2_1_/c, the ligand system adopts a cisoid conformation that allows both the nitrogen centers N1 and N2 (N3 and N4) to coordinate to the cobalt atom. The metal-nitrogen bond lengths are 2.181 Å (Co1–N1) and 2.089 Å (Co1–N2), and the Co–Cl bond lengths are 2.2769 Å (Co1–Cl1) and 2.4428 Å (Co1–Cl2). In complex **4a**, the cobalt atom has distorted tetrahedral coordination geometry with N1–Co1–N2 angle of 77.69° and Cl1–Co1–Cl2 angle of 96.61°. The 2,6-diisopropylphenyl substituent at the imine nitrogen atom is almost perpendicular to the plane of the pyridine moiety (angle between the planes: 89.84°). The angle of N2–C5–C6 is 120.4° when there is a H atom in the imino bridge. Crystallizing in a monoclinic space group P2_1_/n and P2_1_/c, complexes **5a** and **7a** with CH_3_ group in the imino bridge have a smaller N2–C7–C5 angle of 116.4° and N2–C6–C5 angle of 116.58° than those in complex **4a**. Meanwhile, they have longer N–Co bond distances (**5a**: 2.0439 Å, 2.0498 Å; **7a**: 2.0667 Å, 2.0514 Å), shorter Co–Cl bond distances (**5a**: 2.2071 Å, 2.2148 Å; **7a**: 2.2079 Å, 2.2235 Å ), and bigger angles of N1–Co1–N2 (**5a**, 80.68°; **7a**, 80.14°) and Cl1–Co1–Cl2 (**5a**, 115.44; **7a**, 113.88) than complex **4a**. The 2,6-diisopropylphenyl substituent at the imine nitrogen atom is also perpendicular to the plane of the pyridine moiety (angle between the planes: **5a**, 89.56°; 7a, 83.97°). However, ligand **L2** and **L3** (H atom at 6-position of the pyridine ring) results in the formation of a chloro-bridged dimer. In complex **2a**, reported in a previous report [22], the Co–N bond distances are 2.093 and 2.123 Å, which are longer than those in **5a** and **7a** but shorter than that in complex **4a**; however, the Co–Cl bond distance exhibits the converse regularity (**4a** > **2a** > **5a** ≈ **7a**). In complex **3a** (Figure 4), with phenyl group in the imino bridge, the coordination geometry of the central metal atom can be best described as distorted trigonal-bipyramidal with two equivalent half molecules in an overall C_2_-symmetric framework. The pyridyl nitrogen atom (N1) and Cl mark the apical centers of the distorted trigonal bipyramid. The pyridine nitrogen (N1) and the other constituent of the chloro-bridge (Cl2) occupy the axial coordination sites. Complex **3a** has a bipyramidal structure most distorted towards a square-based pyramid, with the most acute angle (164.04°) in the vertical axis (N1–Co1–Cl2) and one of the equatorial angles extended to 125.21°.

**Figure 5:**
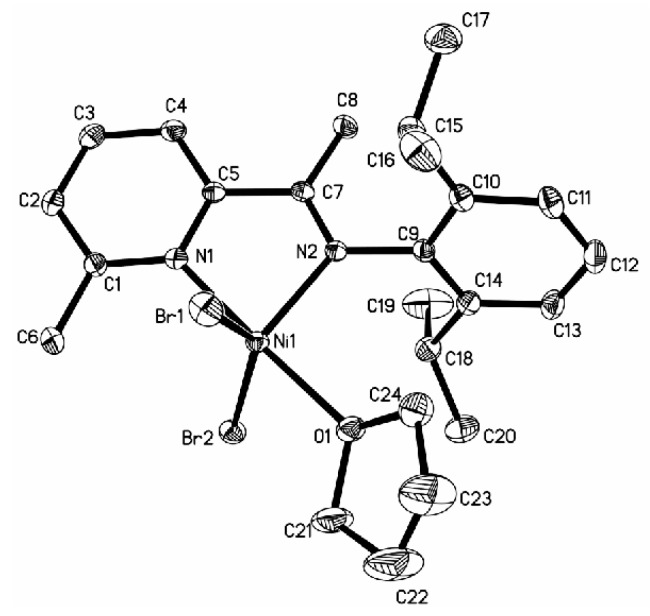
ORTEP view of complex **5b**, drawn at 35% of probability. Hydrogen atoms and one **THF** molecule were omitted for clarity.

**Figure 6:**
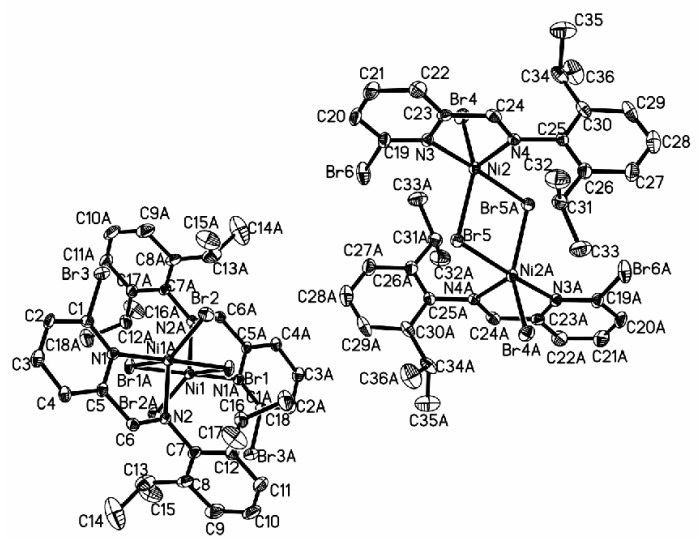
ORTEP view of complex **6b**, drawn at 35% of probability. Hydrogen atoms were omitted for clarity.

Nickel complex **5b** crystallized from THF in a monoclinic space group P2_1_/c, producing the solid structures with a single ligand and one THF molecule coordinated to the metal center. In complex **5b** **(**Figure 5**)**, the nickel is five coordinate, and the coordination sphere could be best described as a distorted trigonal bypyramidal with the equatorial plane occupied by the imino N atom and the two bromine atoms. The axial positions are coordinated by the pyridine N atom and by the O atom of the THF molecule. A Br–Ni–Br angle of 141.13° was observed within the equatorial plane and a “chelating” angle of N1–Ni–N2 is 80.22°. The distance of Ni–O bond is 2.148 Å, which is a little longer than those of the N–Ni bond (2.0383 Å, 2.0677 Å), indicating a very strongly bound THF ligand. Complex **6b** **(**Figure 6**)** with Br at the 6-position of the pyridine ring crystallizes as centrosymmetric dimmers with two ligand nitrogen atoms, one terminal bromine and two bridging bromine atoms forming the coordination sphere around the five-coordinate nickel center. Complex **6b** contains the halves of two independent and nearly identical molecules, which is similar to complex **4b** reported in the literature in that both have nearly the same Co–N bond distance (**4b**: 2.086, 2.042 Å; **6b**: 2.092, 2.049 Å), Ni–Br bond distance (**4b**: 2.5411, 2.4136 Å; **6b**: 2.4214, 2.4777 Å), and N–Ni–N angle (**4b**: 80.29°; **6b**: 80.27°).

### 3.3. Solution Polymerization of 1,3-Butadiene

Cobalt complexes could polymerize 1,3-butadiene activated by ethylaluminum sesquichloride (EASC). Herein, we mainly discuss the influences of the R_1_ group (H, CH_3_ and Br) at the 6-position of the pyridine ring and R_2_ group (H, aldimine; CH_3_, ketimine) in the imino bridge on catalytic activity and selectivity of the complexes. The polymerization results are summarized in Table 4.

**Table 4:** Polymerization of 1,3-butadiene with Co (II) complex/EASC catalyst ^a^.

Run	Complex	Yield (%)	*M*_n_^b^ × 10^−4^	*M*_w_/*M*_n_^b^	Microstructure ^c^ (%)		
					*Cis*-1,4	1.2	*Trans*-1,4
1	**1a**	56.9	23.0	2.4	98.1	1.0	0.9
2	**2a**	51.6	27.7	2.1	98.2	1.0	0.8
3	**3a**	92.3	9.0	3.3	96.5	1.6	1.9
4	**4a**	42.9	31.5	1.9	98.3	0.9	0.8
5	**5a**	31.9	33.8	1.7	98.5	0.9	0.6
6	**6a**	92.8	10.9	3.0	97.2	1.4	1.4
7	**7a**	90.8	12.0	3.2	97.0	1.4	1.6

^a^ Polymerization in hexane at 20 °C for 15 min, [Bd] = 1.85 mol/L, [BD]/[Co] = 1000, [Al]/[Co] = 50. ^b^ Determined by GPC (THF, PSt calibration). ^c^ The microstructure was determined by FTIR.

The substituent (R_1_) at the 6-position of the pyridine ring significantly influence the catalytic performances of the complexes. For the aldimine- and ketimine-based cobalt complexes, the introduction of halogen atom (Br) at 6-position of the pyridine ring afforded polymers in higher yields but with relatively lower molecular weight than the complexes without substituent. The complexes with CH_3_ group produced polymers in lower yield but with higher molecular weight and narrower molecular weight distribution. For instance, the polymerization of 1,3-butadiene with aldimine cobalt complex **6a** (R_1_ = Br) produced polymer in 92.8% yield, which is much higher than complexes **1a** (R_1_ = H, 56.9%) and **4a** (R_1_ = CH_3_, 42.9%). Similar to aldimine cobalt complexes, ketimine cobalt complex **7a** (R_1_=Br) displayed higher catalyst activity (polymer yield: 90.8%) than **2a** (R_1_ = H, 51.6%) and **5a** (R_1_ = CH_3_, 31.9%). On the other hand, the molecular weights of polybutadienes obtained by these cobalt complexes exhibited the converse regularity (in the orders of **6a** < **1a** < **4a** and **7a** < **2a** < **5a**). These results could be explained by the idea that the electron-withdrawing group (Br) at the 6-position reduces the electron density of central metal, and the increased Lewis acidic character facilitates the coordination of 1,3-butadiene molecule, leading to the increment of chain propagation rate. Moreover, due to the electron-contributing and steric hindrance of the CH_3_ group, the coordination reaction between 1,3-butadiene and the cobalt center was retarded [10] . The CH_3_ group can serve to raise polymer molecular weight (**4a**, 31.5 × 10^4^; **5a**, 33.8 × 10^4^) and regulate molecular weight distribution (**4a**, 1.9; **5a**, 1.7), suggesting that electron-contributing substituent at 6-position of the pyridine ring retard chain transfer reaction to some extent.

The substituent (R_2_) in the imino bridge of the iminopyridine cobalt(II) complex was also investigated. Complex **3a** with a phenyl group exhibited much higher activity than **1a** (R_2_ = H) and **2a** (R_2_ = CH_3_). This result indicates that the conjugation of phenyl and imine groups can stabilize the active centers and cause the increased chain propagation rate. In olefin catalysts based on bisiminopyridine transition metal complexes, replacing a ketimine (R_2_ = CH_3_) with an aldimine (R_1_=H) usually leads to difference in molecular weight and molecular weight distribution, particularly the productivity, and usually the catalysts based on an ketimine ligand are approximately one order of magnitude more active than those based on an aldimine ligand [25]. In 1,3-butadiene polymerization, bisiminopyridine cobalt complexes based on ketimine ligand also showed higher catalytic activity than their aldimine counterparts [9]. In the present study, however, iminopyridine cobalt(II) complexes based on aldimine produced the polybutadiene in a higher polymer yield, smaller molecular weight and broader molecular weight distribution than the ketimine complexes, and the results were as follows: **1a** (R_1_=H, 56.9%) > **2a** (R_1_ = H, 51.6%), **4a** (R_1_ = CH_3_, 42.9%) > **5a** (R_1_ = CH_3_, 31.9%) and **6a** (R_1_ = Br, 92.8%) > **7a** (R_1_ = Br, 90.8%). This is due to the fact that, during the activation with EASC, the –CH=N groups of the complexes can be deprotonated to yield an anionic amide ligand. This anionic amide ligand is in the form of an ion pair or in the free form, resulting in higher polymerization activity [12,26].

The polybutadienes obtained by these iminopyridine-based cobalt(II) complexes had high *cis*-1,4 content greater than 96%. Complexes **4a** and **5a** with CH_3_ at 6-position of the pyridine ring produced polymers with higher *cis*-1,4 content (**4a**, 98.3%; **5a**, 98.5%) than the substituent-free (**1a**, 98.1%; **2a**, 98.2%; **3a**, 96.5%) and Br-containing complexes (**6a**, 97.2%; **7a**, 97.0%). However, R_2_ groups in the imino of iminopyridine cobalt(II) complexes did not influence selectivity significantly.

**Figure 7:**
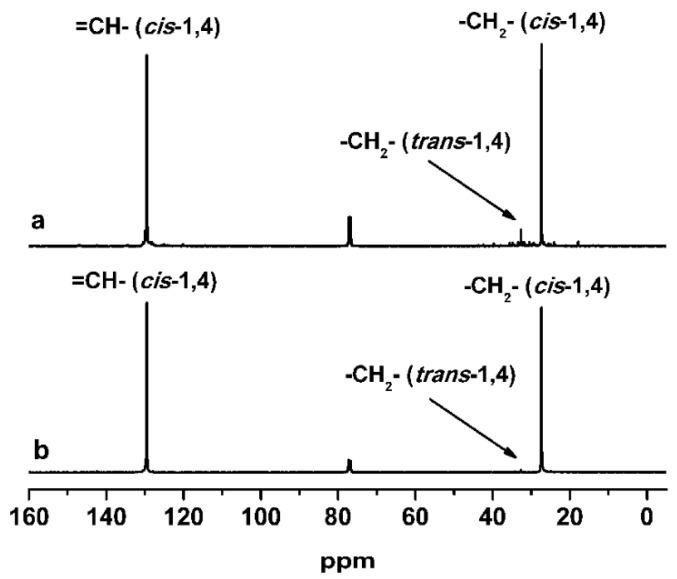
^13^C NMR spectra of polybutadienes obtained by **6a/EASC** (**a**) and **6b/EASC** (**b**).

The nature of the metal center had a large influence on the catalytic performance. In general, cobalt-based catalysts were more active than the corresponding nickel-based analogs; under the same conditions, except for a prolonged polymerization time by 3h employed here, iminopyridine-based nickel complexes produced polymers in 29.2% to 79.3% yields. All the nickel complexes **1b**–**7b** yielded polymers with much lower molecular weights (7000–9900 g/mol), narrower molecular weight distributions (1.72–2.67), and lower *cis*-1,4 contents (79.3%–90.7%) than their cobalt counterparts (Entry 8–14 in Table 4). Similarly, as for the nickel complexes, the aldimine (R_2_ = H) complexes showed higher catalyst activity than the corresponding ketimine (R_2_ = CH_3_) complexes in the orders: **1b** (R_1_ = H, 69.7% conv.) > **2b** (R_1_ = H, 35.8% conv.), **4b** (R_1_ = CH_3_, 75.4% conv.) > **5b** (R_1_ = CH_3_, 47.1% conv.) and **6b** (R_1_ = Br, 79.3% conv.) > **7b** (R_1_ = Br, 72.8% conv.). Moreover, nickel complexes (**6b**, **7b**) with Br at 6-position of pyridine ring exhibited a higher catalyst activity than the cobalt complexes described. However, **3b** with phenyl in the imino bridge showed the lowest catalyst activity, quite different from **3a** with the same ligand, indicating that phenyl group in the complex could retard the chain propagation and chain transfer reaction to produce the polymer with lower yield, higher molecular weight, and narrower molecular weight distribution (Entry 10 in Table 5). The results in Table 5 demonstrated that the ligand environment did not significantly influence molecular weight and molecular weight distribution of the obtained polymers, but remarkably influenced the stereoregularity of the polybutadienes. The aldimine complexes produced polymers with lower *cis*-1,4 content, more *trans*-1,4 content and nearly invariable 1,2 content compared with ketimine complexes. The ^13^C NMR spectra of the polymers obtained with **6a** and **6b** are shown in Figure 7. It is clear that the nickel complex produced the polymers with higher *trans*-1,4 content than the cobalt complex sharing the same corresponding ligand **L6**.

**Table 5:** Polymerization of 1,3-butadiene with Ni (II) complexes ^a^.

Run	Complex	Yield (%)	*M*_n_^b^	*M*_w_/*M*_n_^b^	Microstructure ^c^ (%)		
					*Cis*-1,4	1.2	*Trans*-1,4
8	**1b**	69.7	8,100	2.40	87.1	3.4	9.5
9	**2b**	35.8	8,700	1.87	91.6	3.5	4.9
10	**3b**	29.2	9,900	1.72	90.7	3.8	5.5
11	**4b**	75.4	7,100	2.38	84.1	1.7	14.2
12	**5b**	47.1	8,500	1.87	90.7	3.3	6.0
13	**6b**	79.3	7,200	2.67	79.3	1.3	19.4
14	**7b**	72.8	7,000	2.17	87.1	2.3	10.6

^a^ Polymerization in hexane at 20 °C for 3 h, [Bd] = 1.85 mol/L, [BD]/[Ni] = 1000, [Al]/[Ni] = 50. ^b^ Determined by GPC (THF, PSt calibration). ^c^ The microstructure was determined by FTIR.

## 4. Conclusions

Structurally well-defined iminopyridine ligated cobalt and nickel complexes were synthesized, and their catalytic behaviors in 1,3-butadiene polymerization were investigated. Activated by EASC, the cobalt complexes exhibited high *cis*-1,4 selectivity (up to 98.5%), affording high molecular weight polybutadienes (*M*_n_ = 9.0–33.8 × 10^4^), while the nickel complexes produced polymers with low molecular weight (*M*_n_ = 7000–9000) and *cis*-1,4 stereoregularity (79.3%–91.6%). In both cases of Co- and Ni-based complexes, the aldimine-based complexes showed higher catalyst activity than their ketimine counterparts.

## Appendix

CCDC numbers 893455(**3a**), 893456(**4a**), 893457(**5a**), 893458(**7a**), 893459(**5b**) and 893460(**6b**) contain the supplementary crystallographic data for this paper. This data can be obtained free of charge from The Cambridge Crystallographic Data Centre via www.ccdc.cam.ac.uk/data_request/cif.
